# P-1578. Impact of a Pharmacist-Driven Carbapenem De-escalation Algorithm for Hospitalized Patients with Urinary Tract Infections

**DOI:** 10.1093/ofid/ofae631.1745

**Published:** 2025-01-29

**Authors:** Timothy Gauthier, Susana Diaz, Kelsey Williams, Erika Dittmar, Jefferson Cua

**Affiliations:** Baptist Health South Florida, Miami, Florida; Baptist Health Homestead Hospital, Miami, Florida; Innoviva Specialty Therapeutics, Miami, Florida; Baptist Hospital of Miami, Miami, Florida; Baptist Health South Florida, Miami, Florida

## Abstract

**Background:**

This study aims to assess the impact of a pharmacist-driven carbapenem de-escalation algorithm on the use of antibiotics in hospitalized patients with urinary tract infections (UTIs).

Carbapenem De-escalation Algorithm

**Figure:**
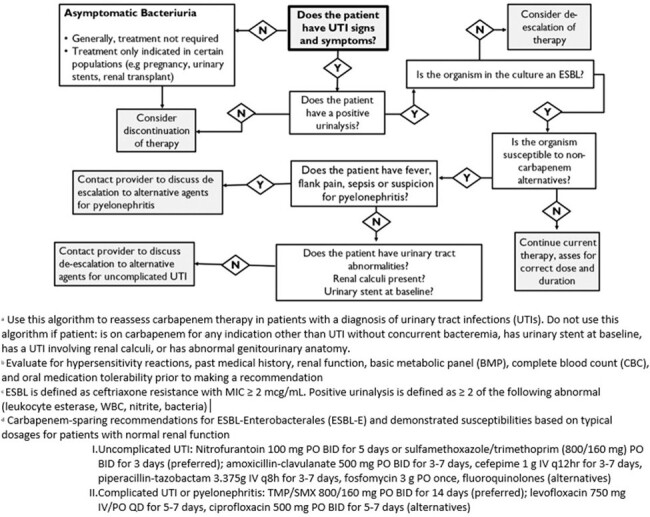

**Methods:**

This was a single-center, retrospective chart review of patients who received carbapenems with a diagnosis of UTIs before (phase I, Aug – Nov 2022) and after (phase II, Dec 2022 – Mar 2023) implementation of a pharmacist-driven carbapenem de-escalation algorithm. Pharmacists made recommendations based on the algorithm but changes to therapy was at the discretion of the ordering provider. Patients aged ≥ 18 years who were receiving carbapenems for the treatment of UTI without concomitant bacteremia were included. Patients who received carbapenems for other indications, had a urinary stent, had abnormal genitourinary anatomy, or presented with renal calculi were excluded. The primary outcome was the percentage of qualifying patients who were de-escalated from carbapenem to non-carbapenem agent before and after the implementation of the algorithm.

Baseline Characteristics

**Figure:**
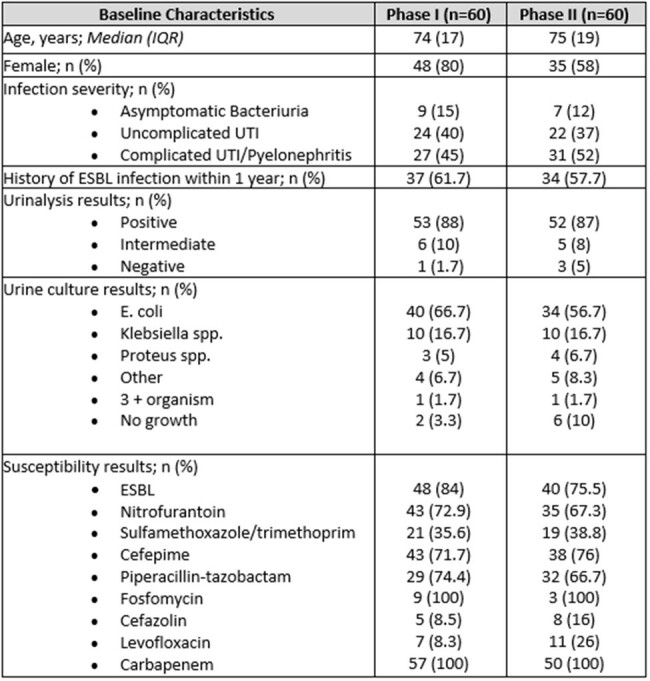

**Results:**

During phase I, 40 of 60 (67%) patients were found eligible for de-escalation based on the algorithm, while 37 of 60 (62%) were eligible during phase II. Table 1 shows demographic data and baseline characteristics. De-escalation occurred in 27 (68%) versus 33 (90%) (95%Cl 0.03 - 0.38) during phase I and II, respectively. De-escalation was attributed to the intervention in 15 (41%) of the phase II group, with a recommendation acceptance rate of 79%. Approximately 35 minutes of pharmacist daily workload was allocated to review and intervene as appropriate. Median duration of carbapenem therapy pre and post intervention was 72 versus 58 hours (p=0.4237) respectively. Median duration of antibiotic therapy in phase I was 7 days, while in phase II it was 6.7 days (p=0.6745).

Results

**Figure:**
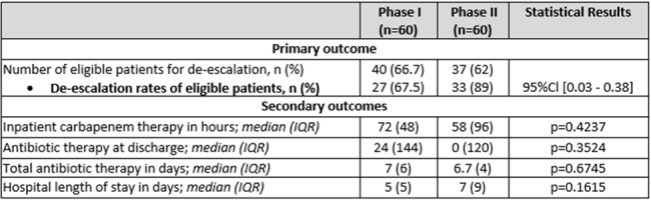

**Conclusion:**

Intervention guided by a pharmacist-driven carbapenem de-escalation algorithm for patients with UTI can expedite carbapenem de-escalation without significantly impacting duration of antibiotic therapy and hospital length of stay.

**Disclosures:**

**Timothy Gauthier, PharmD, BCPS, BCIDP**, AbbVie Pharma: Advisor/Consultant|Antimicrobial Therapy, Inc: Advisor/Consultant|Ferring Pharma: Advisor/Consultant|Firstline Mobile Health: Advisor/Consultant|Gilead Pharma: Advisor/Consultant|GoodRx: Advisor/Consultant|GSK Pharma: Advisor/Consultant|Melinta Pharma: Advisor/Consultant|Pattern Biosciences: Advisor/Consultant|Pfizer Pharma: Advisor/Consultant|ProCE: Honoraria|WWW.LearnAntibiotics.com: Ownership Interest

